# On the Impact of the Degree of Fluorination on the ORR Limiting Processes within Iron Based Catalysts: A Model Study on Symmetrical Films of Barium Ferrate

**DOI:** 10.3390/ma13112532

**Published:** 2020-06-03

**Authors:** Stephan Wollstadt, Oliver Clemens

**Affiliations:** 1 Fachgebiet Materialdesign Durch Synthese, Institut für Materialwissenschaft, Technical University of Darmstadt, 64287 Darmstadt, Germany; wollstadt@md.tu-darmstadt.de; 2 Institut für Nanotechnologie, Karlsruher Institut für Technologie, Hermann-von-Helmholtz-Platz 1, 76344 Eggenstein Leopoldshafen, Germany

**Keywords:** thin film fluorination, ORR catalysts, oxyfluorides, barium ferrate, impedance spectroscopy

## Abstract

In this study, symmetrical films of BaFeO_2.67_, BaFeO_2.33_F_0.33_ and BaFeO_2_F were synthesized and the oxygen uptake and conduction was investigated by high temperature impedance spectroscopy under an oxygen atmosphere. The data were analyzed on the basis of an impedance model designed for highly porous mixed ionic electronic conducting (MIEC) electrodes. Variable temperature X-ray diffraction experiments were utilized to estimate the stability window of the oxyfluoride compounds, which yielded a degradation temperature for BaFeO_2.33_F_0.33_ of 590 °C and a decomposition temperature for BaFeO_2_F of 710 °C. The impedance study revealed a significant change of the catalytic behavior in dependency of the fluorine content. BaFeO_2.67_ revealed a bulk-diffusion limited process, while BaFeO_2.33_F_0.33_ appeared to exhibit a fast bulk diffusion and a utilization region δ larger than the electrode thickness L (8 μm). In contrast, BaFeO_2_F showed very area specific resistances due to the lack of oxygen vacancies. The activation energy for the uptake and conduction process of oxygen was found to be 0.07/0.29 eV (temperature range-dependent), 0.33 eV and 0.67 eV for BaFeO_2.67_, BaFeO_2.33_F_0.33_ and BaFeO_2_F, respectively.

## 1. Introduction

The currently rising awareness for the future need of clean and green energy increases the demand for energy storage and battery applications. Already existing systems like the well-known lithium ion batteries (LIB) and fuel cell based power sources experience an increase in interest. However, struggling with limited power output or high operating temperatures, these systems have to be revised and improved further to cope with future high-performance demands.

Nowadays fuel cell systems are based on several types of archetypes, including alkaline fuel cells (AFCs), phosphoric acid fuel cells (PAFCs), polymer electrolyte membrane fuel cells (PEMFCs), molten carbonate fuel cells (MCFCs) and solid oxide fuel cells (SOFCs). While PAFCs and PEMFCs require precious metals for their operability at low temperatures and AFCs, PAFCs and MCFCs suffer from corrosion problems due to their liquid electrolytes, SOFCs offer the cleanest and most efficient way to transform chemical into electrical energy [1]. SOFCs are characterized by all-solid components and hence, mostly high operating temperatures (>800 °C). However, the high operating temperature leads to cheaper building materials for electrolytes and electrodes such as perovskite type oxides. The high temperature can be an advantage in terms of performance; therefore, the focus on nowadays research lies on reducing it to intermediate temperatures of 500–700 °C, to limit disadvantageous reactions between cell components and thus, prolonging the cell lifetime. Current SOFCs are built of yttria-stabilized zirconia (YSZ) electrolyte, a La_0.8_Sr_0.2_MnO_3_ (LSM) perovskite type cathode and a nickel–YSZ cermet anode [1,2,3]. A critical reaction in such fuel cells is the reduction of oxygen to oxide ions (oxygen reduction reaction, ORR), which requires the transfer of found electrons and is therefore a complex catalytic step [4]. For this reaction, a simple reduction of the operating temperature leads to an increase in cell polarization resistance due to the lack of activation energy [5,6]. Attempts are made by the introduction of stacking multiple materials to profit from their specific characteristics [7] but also by utilizing nanoscale designs [8]. Further, the catalytic activity of the cathode is highly composition dependent, and tremendous efforts have been made to adopt the composition of the perovskite type catalyst by changing the La/Sr ratio or replacing Mn by other metals such as Fe or Co, both showing a strong impact on the average transition metal oxidation state. Recently, also doping of the anion sublattice of the cathode material was targeted [3,9]. In this case, the introduction of anions alters the electronic structure, which directly affects the surface oxygen exchange and the oxygen bulk diffusion [10,11]. Possible anionic dopants are F^−^ and Cl^−^ which reportedly increase the catalytic performance of perovskite oxides [12,13].

For anion doping with fluoride, it is important to keep in mind that most of the perovskite type oxyfluoride materials are only metastable, and decompose into alkaline earth fluorides and transition metal oxides at temperatures between 400–800 °C [3,14]. In addition, it is normally not trivial to monitor the detailed fluorine content easily, since O^2−^ and F^−^ are indistinguishable within the commonly used powder diffraction methods [15,16,17]. The combination of difficult preparation, limited understanding of detailed compositional stability and challenging characterization of materials requires the analysis of systems with well-characterized fluorination behavior in order to develop basic knowledge on general structure-composition-property relationships.

Therefore, the aim of this study is to understand and elaborate the impact of the fluorine content and anion composition on a model system which is known to possess differently well characterized non-fluorinated and fluorinated phases with distinct fluorine contents. To do so, we target the system BaFeO_x_F_y_, for which the phases BaFeO_2.5_ (Ba_2_Fe_2_O_5_, [18]), BaFeO_2.67_ (Ba_3_Fe_3_O_8_*, article in preparation)*, BaFeO_2.33_F_0.33_ (Ba_3_Fe_3_O_7_F [19,20]) and BaFeO_2_F [21,22] are known. The structures of these compounds can all be derived from the cubic perovskite type structure (aristotype); they distinguish by the detailed anion content, which leads to different distortion and ordering variants which are well distinguishable from the observed reflection splitting and superstructure reflections by powder diffraction. We emphasize already at this state the selection of these compounds is not based on high performance criteria since anion ordering is known for a potential negative impact on oxygen ion diffusion [23,24,25], but only on the fact of their well-known fluorination chemistry. Compounds of composition of, e.g., Ba_0.5_Sr_0.5_Fe_1-x_Co_x_O_3−y_F_z_ might be more suitable for their overall electrocatalytic activities, but are only poorly understood with respect to their fluorination behavior. In order to make the reader aware with the manifold phases found in the barium ferrate system and their structural and compositional behavior, these aspects will be summarized in the following:

BaFeO_2.5_ (BFO) crystallizes in a vacancy ordered 28-fold supercell of the cubic perovskite structure with space group *P*2_1_*/c* [18]. The adaption of this monoclinic structure is highly dependent on the anion composition, and can only be found for compositions close to BaFeO_2.5+d_ with d < 0.01 [26]. BaFeO_2.5_ can be prepared by spray pyrolysis approaches [27,28]; this method is known to provide powder morphologies for perovskite-type compounds which are suitable to prepare symmetrical films [8] on 8 mol.%-yttria-stabilized zirconia button substrates (YSZ8), serving as the solid electrolyte. Only recently, we found that this phase can be topochemically oxidized to BaFeO_2.67_ (BFO’, space group *P*2_1_/*m*) at 250 °C (*article in preparation*). Further, BFO can form two different oxyfluoride phases, BaFeO_2_F (BFOF, space group *Pm*3*m*) or BaFeO_2.33_F_0.33_ (BFOF33, space group *P*2_1_/*m*) on topochemical fluorination, which have already been well understood structurally and compositionally [19,22]. For fluorine contents 2x between 1/3 and 1 in the system BaFeO_2.5−x_F_2x_, two phase mixtures were observed [19]. BaFeO_2.67_ and BaFeO_2.33_F_0.33_ are isotypic, but show a significantly difference in unit cell volume, which makes them well distinguishable, and also allows to approximate potential intermediate compositions according to BaFeO_2.67−z_F_z_ (0 ≤ z ≤ 1/3) from the change of lattice parameters.

Further, since these compounds have only limited stability, it is not possible to use such oxyfluoride powders to sinter them into symmetrical films. Therefore, thin film suitable vapor transport related fluorination methods [29,30] are used for the preparation of symmetrical films of BaFeO_2_F, and extended by a newly developed interdiffusion approach for the preparation of symmetrical films of BaFeO_2.33_F_0.33_.

**Figure 1:**
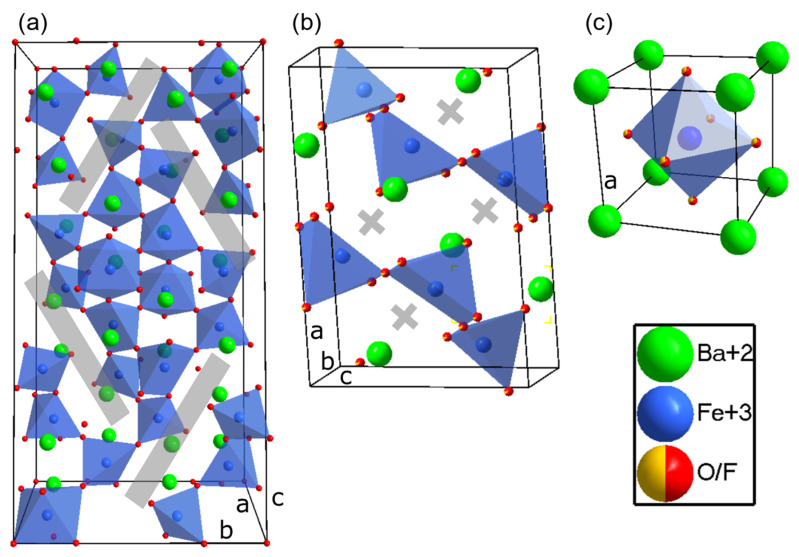
Defect structure of (**a**) BaFeO_2.5_ (*P*2_1_*/c*) and (**b**) BaFeO_2.67_/BaFeO_2.33_F_0.33_ (*P*2_1_/*m*) with bars/crosses indicating oxygen vacancy channels/relaxations [18,19], (**c**) depicts a defect free structure of BaFeO_2_F (*Pm*3*m*). Though BaFeO_2.67_ is confirmed to have the same overall anion content BaFeX_2.67_ than BaFeO_2.33_F_0.33_, neutron diffraction studies are yet needed to confirm the identical vacancy ordering pattern within the √6 × √2 × √3 monoclinic superstructure of the cubic perovskite modification.

All the compounds differ with respect to their level of anion vacancies (represented as ▯ in the following) starting with the highest concentration BaFeO_2.5_▯_0.5_ (*P*2_1_*/c*), BaFeO_2.67_▯_0.33_ (*P*2_1_*/m*), BaFeO_2.33_F_0.33_▯_0.34_ (*P*2_1_*/m*) and BaFeO_2_F_1_▯_0_ (*Pm*3*m*), with ▯ representing an oxygen vacancy, as well as the average iron oxidation state (Fe^3+^ for BFO, BFOF33 and BFOF, Fe^3.33+^ for BFO’). The oxidation states of iron were formerly investigated in [18,19,31] by Mößbauer spectroscopy and were found to be 3+ only for BFO and its fluorinated counterparts. The oxidation state of BFO’ is 3.33+ in average, and has been confirmed by Mößbauer as well as thermogravimetric studies (*article in preparation*). Figure 1 depicts the different structures highlighting the defect structure of the oxygen vacancies. Figure 1a shows the vacancy channels of BFO which would suggest a high oxygen ion mobility, however, due to the oxygen vacancy ordering, oxygen ions are highly immobile [18]. On the other hand, BFOF33 exhibits a different channel like vacancy ordering in Figure 1b, while BFOF (Figure 1c) is free of defects. It might be noteworthy that hitherto the defect structure of BFO’ has not been investigated yet. Nevertheless, it is likely to be highly similar to BFOF33 due to the identical space group and super cell; however, the distribution of anions on the different anion sites needs to be confirmed by neutron diffraction experiments, but is of subordinate importance for the present study. The isotypic nature of BaFeO_2.67_ and BaFeO_2.33_F_0.33_ could give an indication for the influence of fluorine incorporation without apparent change of the symmetry. In summary, the compounds are able to separate the influences of parameters important for the ORR, such as iron oxidation state, filling degree of the anion lattice and a potential impact of the fluoride ion itself due to its higher mobility than an oxide ion. This is elaborated in detail within this article by a combination of powder diffraction studies with temperature dependent impedance spectroscopy on symmetrical films on YSZ8. It was found that the data can be analyzed on the basis of impedance models describing the charge-transfer and non-charge-transfer process of a porous mixed ionic electronic conducting (MIEC) cathode, and that the filling degree of the anion lattice has indeed a major impact on the electrocatalytic properties of the samples.

## 2. Experimental

### 2.1. Sample Preparation

**Figure 2:**
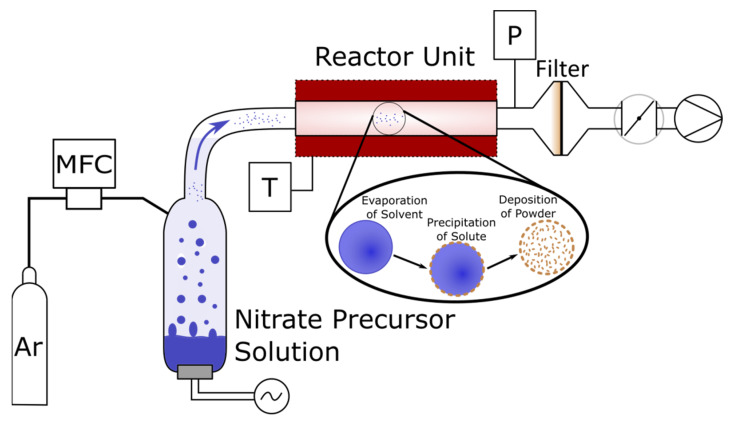
NSP experimental setup with the schematical precipitation process.

Nanocrystalline powder of BaFeO_2.5_ (BFO) was synthesized using the nebulized spray pyrolysis (NSP) method, see Figure 2. The aqueous precursor solution contained the nitrate salts of the corresponding metals with a concentration of c(Ba^2+^) = c(Fe^3+^) = 0.06 mol/L. For that, Ba(NO_3_)_2_ (Sigma Aldrich, Saint Louis, MO, USA, 99.99%) and Fe(NO_3_)_3_ 9 H_2_O (Sigma Aldrich, Saint Louis, MO, USA, 99.99%) were weighed in the stoichiometric ratio and mixed with deionized water. The as-prepared solution was then stirred for 1 h to dissolve the salts and to form a homogenous transparent solution. The yellowish solution was nebulized inside the nebulizer unit with an ultrasonic nebulizer membrane (TDK, NB-59S-09S-0, Tokyo, Japan), which was operated at a voltage of 48 V and 0.5 mA. The mist was then carried with a constant gas stream of argon with a flow rate of 2.5 SLM (standard liter per minute) through the hot wall reactor, held at a constant temperature of 700 °C. The pressure inside the setup was kept at 900 mbar applied by a membrane pump and controlled by a butterfly valve. The precursor solution level was kept constant with a syringe pump at a rate of 1.3 mL/min. The as-synthesized powder was collected on a filter, which was kept at a temperature of 130 °C in order to prevent any condensation of water. The collected powder was then calcined in an airtight tube furnace at a temperature of 900 °C for 20 h under a constant flow of argon to achieve formation of phase pure, water-free BaFeO_2.5_. Another identical powder was prepared at a reactor temperature of 1050 °C. The preparation of two different powders is used to emphasize the influence of the synthesis parameters onto the product powder. Previous reported synthesis approaches rely on a salt-assisted approach where the addition of NaCl to the precursor solution leads to a fine grained morphology of the product. The use of NaCl in the precursor solution of BaFeO_2.5_ resulted in the formation of chlorine containing hexagonal perovskites [32], which are structurally not easy comparable to the compounds under study here.

Symmetrical oxidic template films were produced by spin-coating a stabilized homogenous dispersion of the as-prepared powder onto YSZ8 button substrates (fuelcellmaterials, Nexceris, Lewis Center, OH, USA) followed by sintering. The homogenous dispersion was prepared by mixing the calcined powder with an acidic solution of diluted HNO_3_ (pH = 4). The stability of the dispersion was investigated by a zeta potential measurement using a ZetaSizer Nano SZ (Malvern Instrument, Malvern, UK). HNO_3_ and NaOH were used to adjust the pH level and NaCl was added to the solution to improve the measurement signal. An ultrasonicator was used to distribute the powder evenly in the acidic solution. The insertion of an interlayer, such as GDC, between YSZ substrate and film was not necessary, since no reaction between YSZ and BaFeO_2.5_ was observed. The as-prepared films where sintered at 1060 °C for 1 h under a steady flow of argon.

**Figure 3:**
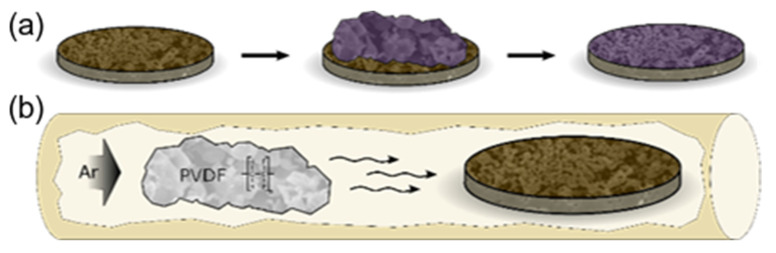
Topochemical fluorination of oxidic films to achieve (**a**) partial fluorinated BaFeO_2.33_F_0.33_|YSZ8|BaFeO_2.33_F_0.33_ via an interdiffusion approach and (**b**) fully fluorinated BaFeO_2_F|YSZ8|BaFeO_2_F via the vapor transport related technique.

The topochemical fluorination of the as-prepared BFO films was done in two different ways to achieve the desired fluorine content in the symmetrical films: BaFeO_2.33_F_0.33_|YSZ8|BaFeO_2.33_F_0.33_ and BaFeO_2_F|YSZ8|BaFeO_2_F. The films were fluorinated either via an interdiffusion or a vapor transport process, respectively; both processes are schematically depicted in Figure 3. The approach of interdiffusion (Figure 3a) allows for controlled fluorine content, whereas the vapor transport related technique (Figure 3b) is less aggressive method for the preparation of high fluorine content phases without decomposition into the thermodynamically stable compounds. The fluorine content of the partial fluorinated BaFeO_2.33_F_0.33_|YSZ8|BaFeO_2.33_F_0.33_ was adjusted by covering the pure oxide films with a sufficient amount of separately prepared BaFeO_2.33_F_0.33_ powder, which serves as a type of infinite reservoir due to its high excess in comparison to the film (the preparation of BaFeO_2.33_F_0.33_ powder is described in [19]). The films and powder were kept at a temperature of 450 °C under argon for 20 h to enable interdiffusion and compositional equilibration between powder and film. In contrast, fully fluorinated BaFeO_2_F|YSZ8|BaFeO_2_F was prepared by utilizing the vapor transport related technique [33]. The films and the polymer polyvinylidene fluoride (PVDF) were put into a tube furnace (Sigma Aldrich, Saint Louis, MO, USA) at an elevated temperature of 450 °C and an argon gas stream. By this, the PVDF is decomposed and decomposition products such as traces of HF are transported over the samples leading to the desired topochemical under formation of the highly fluorinated state.

We would like to emphasize that the use of an inert atmosphere during the preparation processes as well as for storage must be ensured, since it is known that BaFeO_2.5_ is prone to attract moisture leading to a basic reaction with atmospheric CO_2_ resulting into the decomposition into BaCO_3_ and BaFe_2_O_4_ [34].

### 2.2. Ambient and Variable Temperature In-Situ Diffraction Experiments

The ambient and variable temperature X-ray diffraction (XRD) experiments were carried out with a Bruker D8 diffractometer (Bruker, Karlsruhe, Germany). The setup uses a Bragg–Brentano geometry, a Cu-K_α_ source and a PSD VANTEC detector. Ambient measurements were carried out over an angular range of 20° to 80° 2θ with a step size of 0.0066° per step and 1 s measurement time per step. For the variable temperature measurements, an HTK 1200 N temperature chamber with a TCU 1000 N (Anton-Paar) was used. The angular range was set from 20° to 60° 2θ with a step size of 0.015 and 0.22 s measurement time per step. Diffraction experiments were carried out at temperatures ranging from room temperature to 800 °C under argon atmosphere. Scans were carried out in a timed interval of 30 min including a ramping with 5 °C/min and an equilibration time of 10 min for an equal temperature distribution inside the sample chamber. For each scan, the obtained data were qualitatively and quantitatively refined using the Rietveld method with the program TOPAS 6 (Bruker AXS, Karlsruhe, Germany) [35].

### 2.3. Scanning Eelectron Microscopy

The scanning electron microscopy (SEM) was carried out on a Philips XL30-FEG (Philips, Amsterdam, the Netherlands) secondary electron microscope with an acceleration voltage of 20 kV. The powder samples and films were sputtered with gold to avoid electric charging of the surface.

### 2.4. High Temperature Impedance Spectroscopy

To investigate the electrical properties of the films, high temperature impedance spectroscopy was used and samples were measured under oxygen. The frequency range was chosen to be from 1 MHz down to 100 mHz in a temperature window of 400 °C to 600 °C with 20 °C steps. Prior to each measurement, the temperature was kept at the desired set point for 15 min for equilibration. Though the temperature is equilibrated, small temperature fluctuations in the order of +/−1 K cannot be avoided in our setup, and cause visible fluctuations in the impedance spectra only in the range of ~10^0^ to 10^−1^ Hz according to our experience. Further, the temperature for the evaluation process was taken from a thermocouple directly mounted near the sample inside the furnace. An AC amplitude of 100 mV was applied. For the measurement, a Solartron 1260 (Ametek, Berwyn, PA, USA) was used. Prior to the measurement, both sides of the films were sputter-coated with a thin gold layer for improved contacting.

## 3. Results and Discussion

### 3.1. Powder Preparation

**Figure 4:**
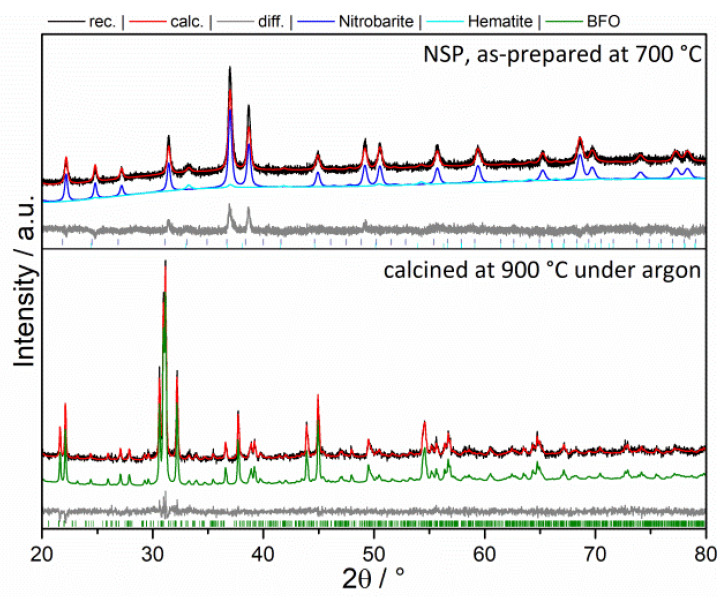
XRD of as-prepared NSP powder and after the calcination step at 900 °C.

The XRD pattern (Figure 4) of the precursor powder obtained via NSP at 700 °C shows a mixture which mainly consisted out of barium nitrate and iron oxide (Fe_2_O_3_). However, after the calcination step at 900 °C, this mixture completely reacted to BaFeO_2.5_ (BFO).

**Figure 5:**
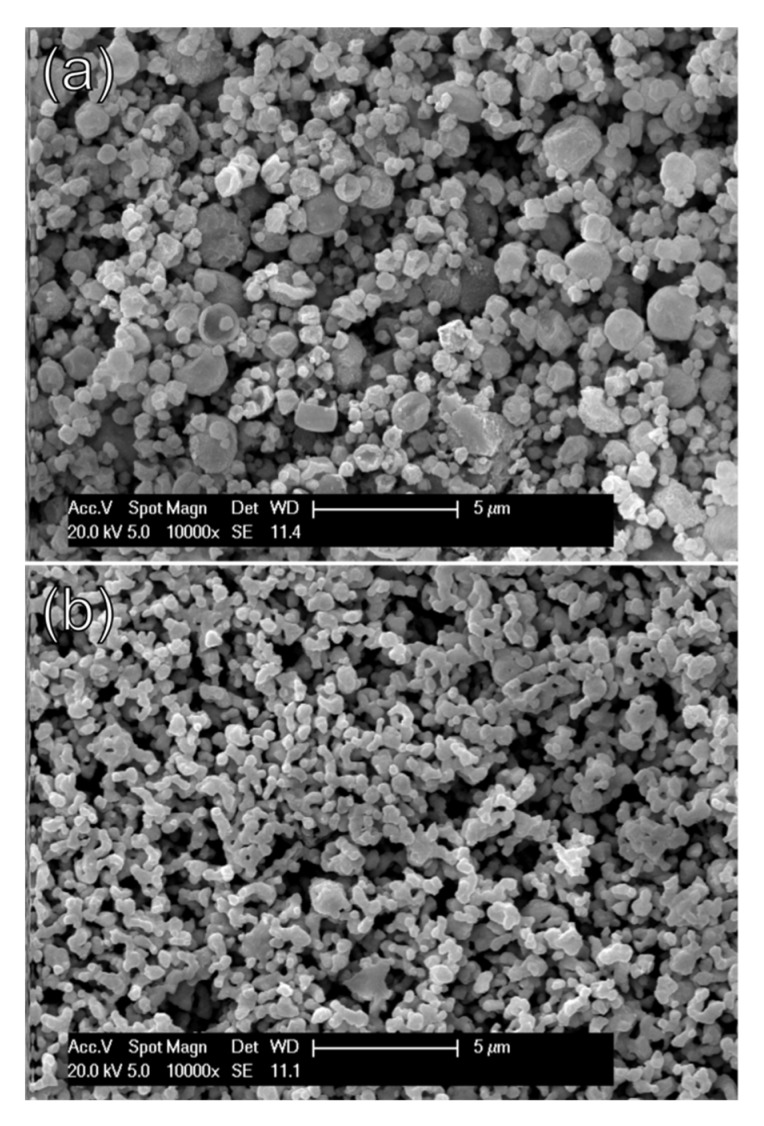
SEM micrographs of (**a**) as-prepared NSP powder at 750 °C and (**b**) after calcination at 900 °C.

The SEM micrographs in Figure 5 show the morphology of as-prepared powder (Figure 5a) as well as (Figure 5b) after the calcination. Right after the synthesis the powder shows the typical hollow sphere morphology [8,36,37,38] resulting from the precipitation at the surface of the nebulized droplets inside the reactor during the process. After the calcination, the hollow-sphere morphology is changed into finely shaped granular particles.

**Figure 6:**
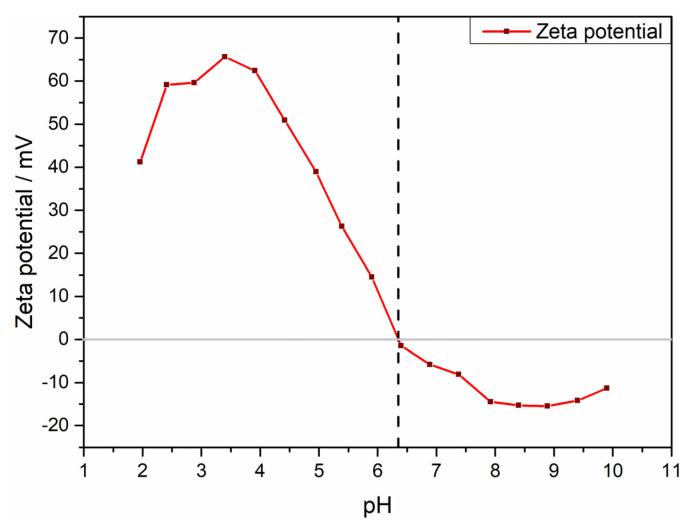
Zeta potential measurement of an aqueous solution of BFO in the pH range from 2–10.

A zeta potential measurement was used to determine the stability of the aqueous dispersion of BFO in deionized water as a function of the pH value. Figure 6 shows the recorded zeta potential curve for the performed measurement. The measurement indicates the highest stability of the dispersed sample material in the acidic region around pH = 3.5, with a maximum zeta potential of ~65 mV. At lower pH, the sample tends to dissolve. Taking this into account, a pH of 4 was used as the best compromise to obtain a stable dispersion in the absence of particle agglomeration.

**Figure 7:**
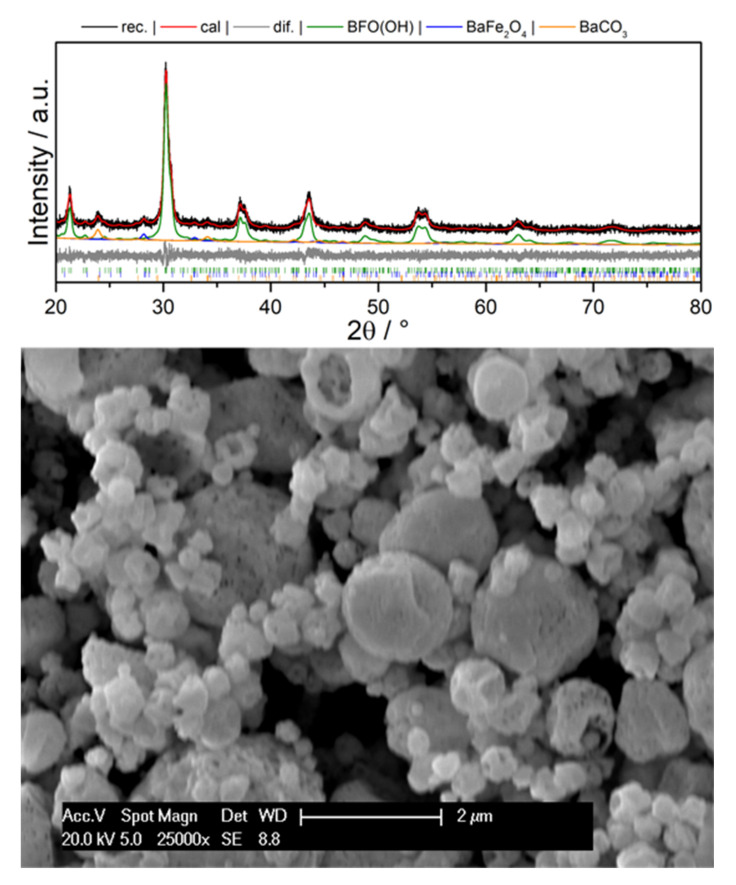
XRD pattern of as-synthesized water-containing BFO and SEM micrograph of the dried BFO.

We would like to emphasize that the reactor unit temperature plays a crucial role for the preparation of BFO precursor powders suitable for the subsequent fabrication of thin films. On increasing the reactor temperature to 1100 °C, a single phase water containing variant of BaFeO_2.5_ (BFO(OH)) is formed [27,39], with some impurities of BaCO_3_ and BaFe_2_O_4_ due to the sensitivity towards CO_2_, see XRD in Figure 7. Upon drying at 450 °C, the compound transforms into monoclinic BaFeO_2.5_. The SEM micrograph of the dried powder (Figure 7) revealed hollow-sphere morphology. It was found that this morphology could not be broken into smaller nanoparticles by ultrasonication due to the sintering process, which already took place inside the reactor. In addition, NaCl assisted approaches which helped to obtain non-agglomerated particles for Ba-free SOFC catalysts were attempted, but resulted in the formation of a hexagonal perovskite type modification [32] due to the incorporation of chloride into the anion lattice. Though similar acidic solutions can be used for the dispersion of hollow-sphere BFO powder, attempts to manufacture symmetrical films via high temperature routes led to unstable films which delaminated easily and could not be characterized further.

### 3.2. Variable Temperature XRD

The metastability of oxyfluorides combined with the intention to investigate these materials for their ORR activity urges the necessity of knowing the decomposition behavior at elevated temperature. It was already shown that BaFeO_2_F decomposes at elevated temperatures into BaF_2_ and BaFe_2_O_4_ [14], however a detailed study of the temperature dependent structural stability was not performed so far. The same holds true for the decomposition of BaFeO_2.33_F_0.33_, for which no data have been reported yet. Hence, variable temperature XRD (VT-XRD) studies were performed to estimate the suitable heating limits, which might also serve as an indication in which temperature range the compounds could in principle be targeted to be used as electrode catalysts in SOFC applications.

**Figure 8:**
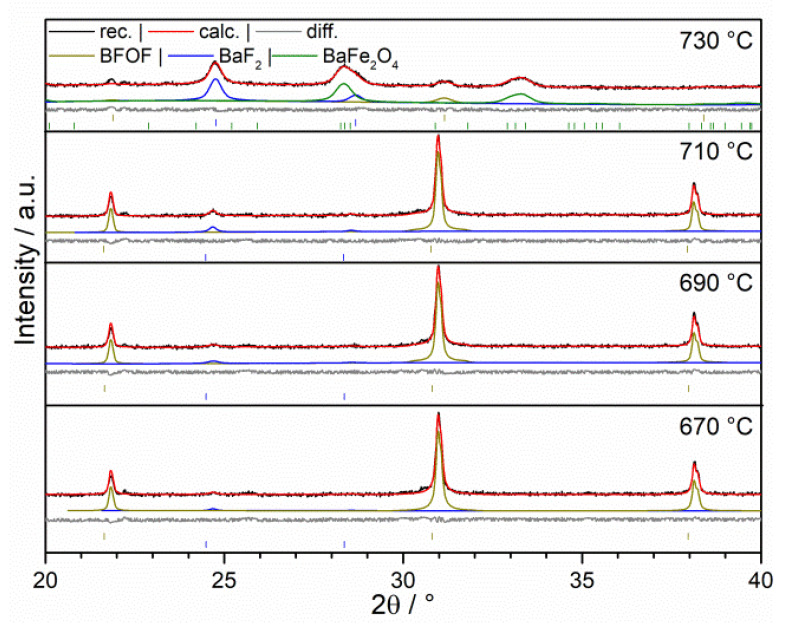
Variable temperature XRD of BaFeO_2_F under argon, 690–730 °C.

**Figure 9:**
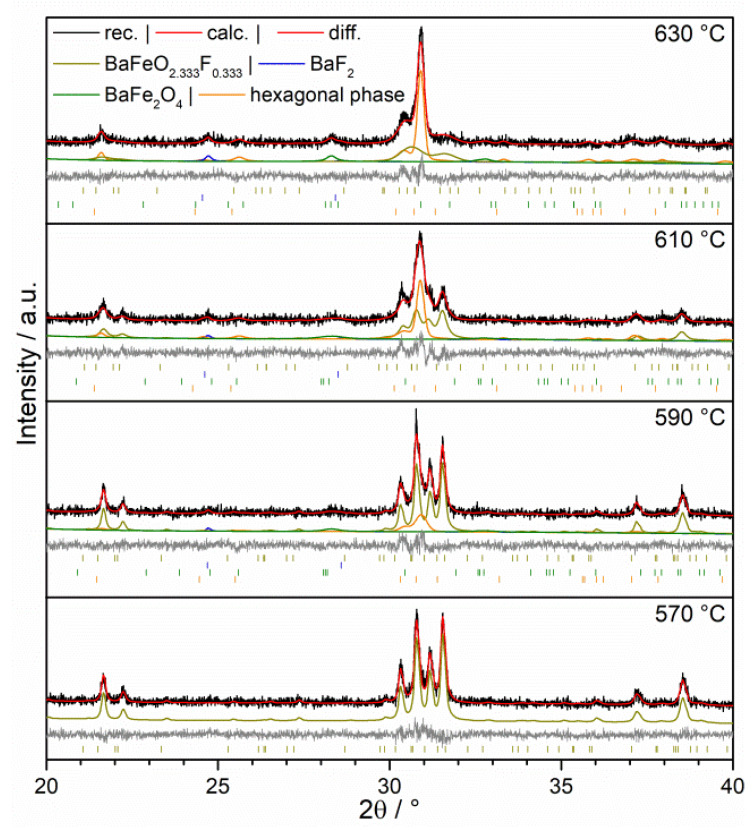
Variable temperature XRD of BaFeO_2.33_F_0.33_ under argon, 570–630 °C.

The results of the VT-XRD studies in Figure 8 for BaFeO_2_F and Figure 9 for BaFeO_2.33_F_0.33_, and representative powder patterns are depicted for the compounds around their respective temperatures of decomposition. For BaFeO_2_F, a first indication for the formation of BaF_2_ (reflex at 24.7° 2θ) was given around 670 °C. Between 670 °C and 710 °C, only a small amount of BaF_2_ (1.45 wt.%) is formed besides a stable main phase of BFOF. At around 710 °C, the amount of this phase increases, and at 730 °C the compound has decomposed into BaF_2_ and BaFe_2_O_4_ according to

2 BaFeO_2_F → BaF_2_ + BaFe_2_O_4_ (1)

As shown previously [14], quantification of the decomposition products for BaFeO_2_F can also serve to confirm the composition of the starting compound, which was also performed here and confirmed the sample to be close to BaFeO_2_F within errors (48.5 mol.% of BaF_2_/51.5 mol.% of BaFe_2_O_4_).

In contrast, BaFeO_2.33_F_0.33_ exhibits an earlier onset of degradation with extended structural changes over a broader temperature range. At 590 °C the XRD shows the formation of a small amount of BaF_2_ combined with the formation of presumably one of the hexagonal perovskite polytypes, which are well known for system BaFeO_3-y__-d_F_y_ (6H, 10H, 12H, 15R) [31,32,40,41,42,43,44,45].

The behaviors on temperature increase of the low fluorine content phase BaFeO_2.33_F_0.33_ (onset around 590 °C) and the high fluorine content phase BaFeO_2_F are partly counterintuitive. In previous works, the metastability of oxyfluorides was mainly attributed to the high stability of the alkaline earth fluorides and lanthanide oxyfluorides. This is certainly true as the general driving force, but it cannot serve to explain the findings shown here. Therefore, the structural stability (transformation between different AX_3_ stacking sequences or formation of vacancy ordered modifications) dominated by the respective lattice energies as a function of fluorine content seems to be more complex than thought, indicating that fluoride richer metastable systems must not necessarily be less stable for the same A/B composition of an ABX_3-d_ perovskite type system. Nevertheless, the findings here indicate that a testing of the oxyfluoride compounds up to 600 °C is experimentally justified.

### 3.3. Film Preparation

**Figure 10:**
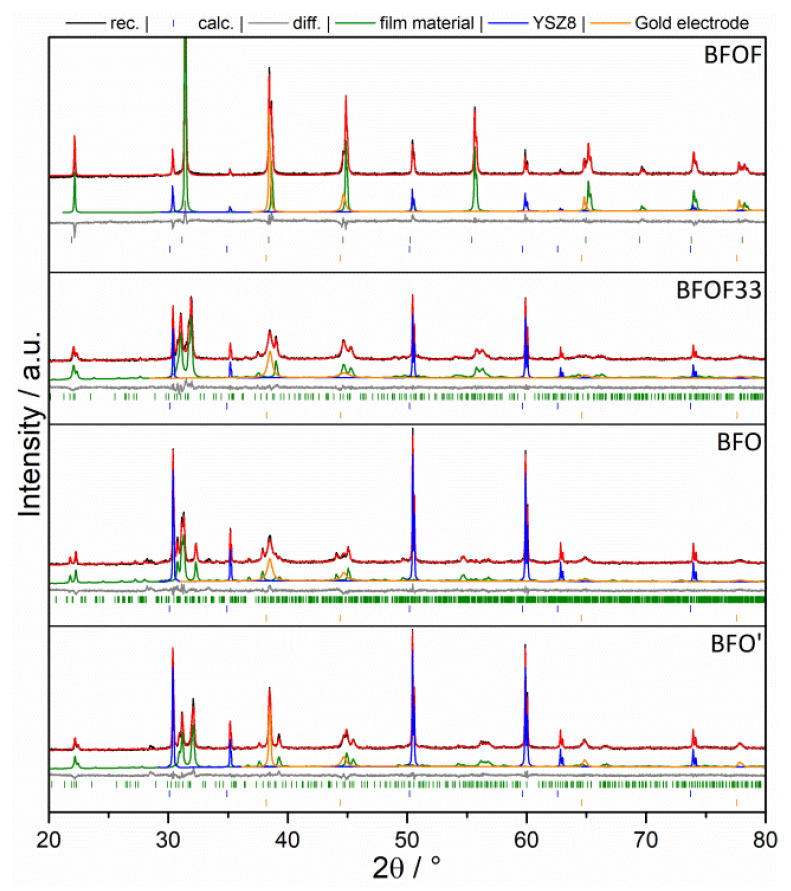
XRD of as-prepared films with sputtered Au electrodes on top after topochemical oxidation and fluorination.

**Figure 11:**
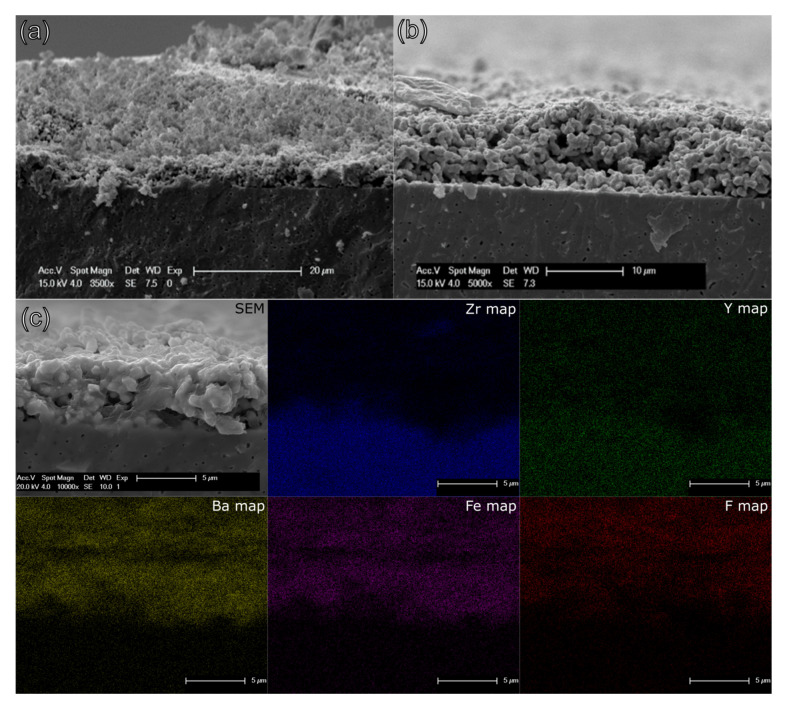
SEM before (**a**) and after (**b**) sintering. EDX (**c**) of a topochemically fluorinated BFOF film, mapping the main elements of the used materials: Zr, Y, Ba, Fe, F.

Figure 10 shows the XRD patterns of sintered BFO films on YSZ8. A Rietveld refinement revealed the presence of exclusively BFO and YSZ8 in both cases. Hence, it is evident that there was no reaction occurring between the substrate material YSZ8 and the film compound BFO (e.g., with formation of BaZrO_3_ [46,47]), further confirmed by the energy dispersive X-ray EDX studies shown later within this article. The formation of phase pure products of BFO’, BFOF and BFOF33 could be confirmed by the diffraction studies (again, see Figure 10). In addition, the cubic YSZ8 substrate (*Fm*3*m*, a = 5.17 Å). This becomes clear on comparing the refined lattice parameters and crystal symmetries (which are known to be highly sensitive on the detailed composition of the anion sublattice [21]) of the compounds within the film to what has been reported in literature (see Table 1). The SEM analysis (Figure 11) reveals a film consisting of a porous network of BFO with a thickness of around 8 μm. The spin coated material, shown in Figure 11a, is already in a network-like arrangement before sintering, which is counterintuitive since the stabilized dispersion should guarantee a densely packed deposition of the spin coated sample powder after the evaporation of the dispersant. After sintering, Figure 11b, the film density is lowered; however, a certain degree of porosity is retained. Since the fluorination reaction is a low-temperature topochemical process (reaction temperature at T ≤ 450 °C), which does not result in grain growth, the EDX in Figure 11c supports the observation made in the XRD pattern, and proves that no diffusion between film and substrate took place, hence, the use of an intermediate layer between film and substrate rendering an intermediate layer unnecessary, such as GDC [39,48,49,50], was not required. No traces of zirconium or yttrium can be found in the film, no barium or iron diffused into the electrolyte and fluorine can only be found in the oxide film and no fluorination of the substrate occurred.

### 3.4. High Temperature Impedance Spectroscopy

**Figure 12:**
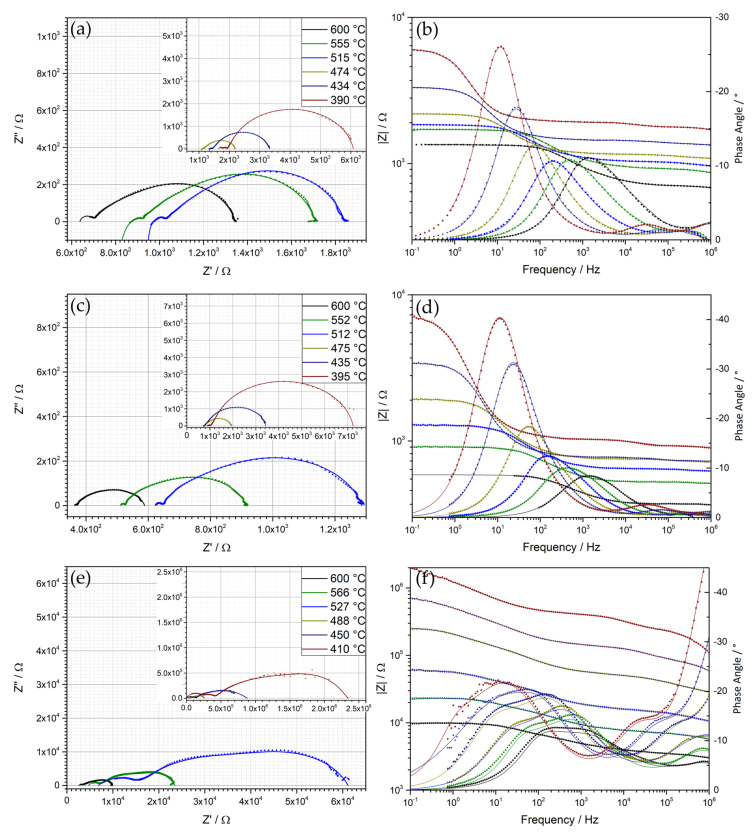
(**a**,**c**,**e**) Nyquist and (**b**,**d**,**f**) Bode plots of symmetrical films of BFO, BFOF33 and BFOF. The recorded data are represented by symbols, while the fits are drawn with solid lines.

Figure 12 depicts the Nyquist and Bode plots of the measured symmetrical films of BFO, BFOF33 and BFOF at selected temperatures in the range of 390 to 600 °C. In this temperature range, the Nyquist plots of BFO (Figure 12a) show a small semicircle at high frequencies, while in the mid frequency range the Nyquist plots first exhibit an open depressed semicircle, which closes between 434 and 450 °C into a depressed semicircle with nearly linear part with a 45° angle on its high frequency side. This mid to low frequency half tear-drop shaped response can generally be described by the Gerischer impedance element [51] and is typically found for porous mixed ionic-electronic conducting oxide cathode materials [52,53,54]. This response is described by:

(2) $$Z_{\mathrm{chem}}=\frac{R_{\mathrm{chem}}}{\sqrt{1+{\left({j\omega t}_{\mathrm{chem}}\right)}^{\varphi }}}$$

with R_chem_ being a chemical resistance associated with non-charge transfer processes inside the electrode, namely oxygen exchange between gas phase and electrode material and oxygen ion diffusion inside the MIEC material, and t_chem_ the corresponding time constant. The exponent φ turns the proposed ideal model into a “fractal” model as discussed in [54], which is comparable to the transformation of a pure capacitive into a constant phase element (CPE) related response. This equivalent circuit element represents an electrochemical non-charge transfer process, which is limited by the transport of oxygen ions with a fast exchange of oxygen at the gas phase and cathode interface. This element is derived from the model developed by Gerischer for the oxygen ion exchange on the surface of a metallic membrane [51]. Adler et al. [52] adapted this model for porous MIEC oxide cathode films, discussing the influences of the overall film morphology onto electrochemical and impedance behavior under the assumption of the electrode characteristics determined by bulk kinetics and transport processes. This Gerischer type model will be used for describing the measured response of the symmetrical BFO films. In order to account for the charge transfer processes occurring in a MIEC cathode, such as electron and oxygen ion exchange at the interfaces between cathode and gold top electrode and cathode and YSZ electrolyte, respectively, the model was extended by two additional R|CPE-elements [55]. We acknowledge that it is not always possible to resolve all processes easily. Often, the Bode plot (e.g., Figure 12b) only shows one broad maximum in the mid to low frequency range and one maximum at high frequencies, leading to the assumption that the non-charge transfer and charge transfer process possess a similar relaxation time. Hence, leading to the following fitting models:

R_S_I-(R|CPE)_1_-(R|CPE)_2_-(R|CPE)_3_ for T < 435 °C (3)

and

R_S_I-(R|CPE)_1_-(R|CPE)_2_-Gsu for T > 435 °C (4)

with Gsu representing the fractal Gerischer model. The indices 1 and 2 represent the electronic and ionic charge transfer processes, respectively [55]. For T < 435 °C, the third circuit (RICPE)_3_ may represent gas phase diffusion inside the porous cathode which then turns into the Gerischer type response at higher temperatures.

In contrast, the response of BFOF33 shown in Figure 12c exhibits also open semicircles up to a temperature of 435 °C, with closed semicircles above this temperature. However, it does not exhibit a 45° angle of the last semi-circle in the temperature range under investigation. Hence, the response appears mainly to be of capacitive nature without a diffusive part as it has been observed in the pure oxide film. The Bode plot in Figure 12d consists of a broad maximum at low to mid frequencies and another one at high frequencies. From this, it was found that a plausible fitting model of a series of (R|CPE) models is sufficient to achieve a satisfying fit:

R_S_-(R|CPE)_1_-(R|CPE)_2_-(R|CPE)_chem_ (5)

**Figure 13:**
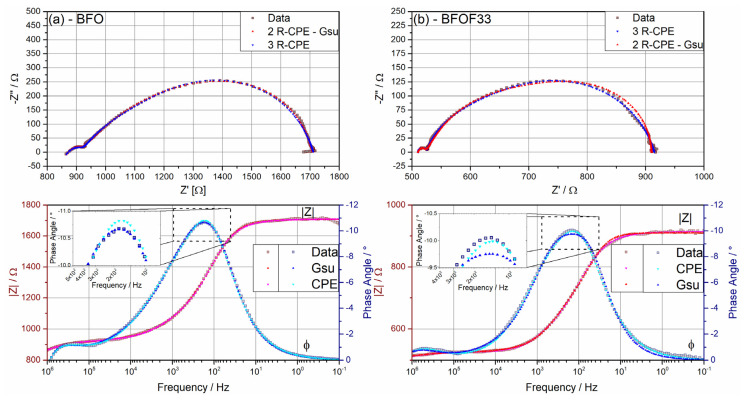
Comparison of fitting models describing diffusive and/or capacitive responses for (**a**) BFO’ and (**b**) BFOF33, where each, (**a**) and (**b**), contain a comparison of the used models inside Nyquist and Bode plots.

This is enforced by testing the models derived for the different compounds on the other compounds (e.g., the model derived for BFO’ on BFOF33). Figure 13 compares the two discussed models on the basis of the obtained impedance data for BFO’ and BFOF33. This comparison shows that

(1) BFO’ can be roughly fitted with the model used for BFO33. However, this series of (R|CPE)_x_ elements only shows significant misfits and deviations in frequency range 10^3^ to 10^1^ Hz, indicating that it does not describe the occurring oxygen exchange at the gas phase/MIEC interface correctly.
(2) In case of BFOF33 (Figure 13b), the (R|CPE)-based fit yields a better result than a Gerischer-based, which again is expressed by systematic misfits in the frequency range of 10^3^ to 10^1^ Hz where the third semicircle expresses most significantly.

BFOF exhibits a different response as shown in the Nyquist and Bode plots, Figure 12e,f. The measured polarization resistance is three orders of magnitude higher than in case of BFO’ and BFOF33. The Bode plot shows two discriminable maxima in the mid to low frequency range. It was noted that in contrast to the semicircles observed for BFO’ and BFOF33, more semicircles are apparent in the high frequency range up to a temperature of 520 °C. On the other side, the Nyquist plots show two semicircles with an open end for temperatures <488 °C in the mid to low frequency range, which show up as a maximum in the frequency representation of the corresponding Bode plots. Following this argumentation, the Nyquist and Bode plots may suggest a simple series of four (R|CPE) circuits. The first two semicircles, apparent only for BFOF, may correspond to a bulk and grain boundary conduction since the capacities are in the order of 10^−12^ and 10^−10^ F, respectively. Due to the comparably high ionic conductivity of YSZ, these resistive contributions must be assigned to the film material, while the contributions of YSZ can be summed up in the low resistive contribution of serial resistor R_S_ as described in the preceding discussions. Hitherto, we described the electrochemical processes inside the MIEC with a 3D model. However, the impedance spectra observed for BFOF do not allow for the application of such a complex model due to the high resistances and less complex spectra observed. Thus, the fitted parameters were reduced to the electrochemical processes to the triple phase boundary (TPB) between electrode, electrolyte and gas phase captured by two serial (R|CPE) elements. We must acknowledge that the fits in the Nyquist and Bode representation still show systematic deviations. However, increasing the number of circuits or using other models, e.g., a Warburg-type element, did either result in over-parametrization and fit divergence or resulted in worse description of the data respectively. Hence, the following models were used for BFOF:

R_s_-(R|CPE)_B_-(R|CPE) _GB_-(R|CPE)_1_-(R|CPE)_2_ for T < 520 °C (6)

and

R_S_-(R|CPE) _GB_-(R|CPE)_1_-(R|CPE)_2_ for T > 520 °C (7)

The impedance spectra recorded relate to the effects of oxygen dissociation at the electrode surface and to the incorporation into the bulk lattice of the electrode material. This can be expressed by the following formalism according to [56]:

(8) $$\mathrm{adsorption}:\;O_{2}{+V}_{O_{s}}^{q_{s}\;}{+q}_{\mathrm{ads}}e^{-}\;⇄{\;(O}_{2}{)}_{O_{s}}^{q_{O_{2}\;}}$$

(9) $$\mathrm{dissociation}:\;{{(O}_{2})}_{O_{s}}^{q_{O_{2}\;}}{+V}_{O_{s}}^{q_{s}\;}{+q}_{\mathrm{diss}}e^{-}\;⇄{\;2O}_{O_{s}}^{q_{O\;}}$$

(10) $$\mathrm{incorporation}:\;O_{O_{s}}^{q_{O\;}}{+V}_{O_{b}}^{∙∙}{+q}_{\mathrm{incorp}}e^{-}\;⇄{\;O}_{O_{b}}^{X}{+V}_{O_{s}}^{q_{s}\;}$$

with b and s representing the corresponding bulk and surface site, respectively. Due to the topochemical nature of the fluorination reactions, which do not enforce any significant morphological changes, microstructural effects must be expected to be low and the differences found between the oxide and oxyfluoride compounds can be assumed to relate to the processes of oxygen transfer and oxygen ion incorporation/release within the symmetrical films.

**Figure 14:**
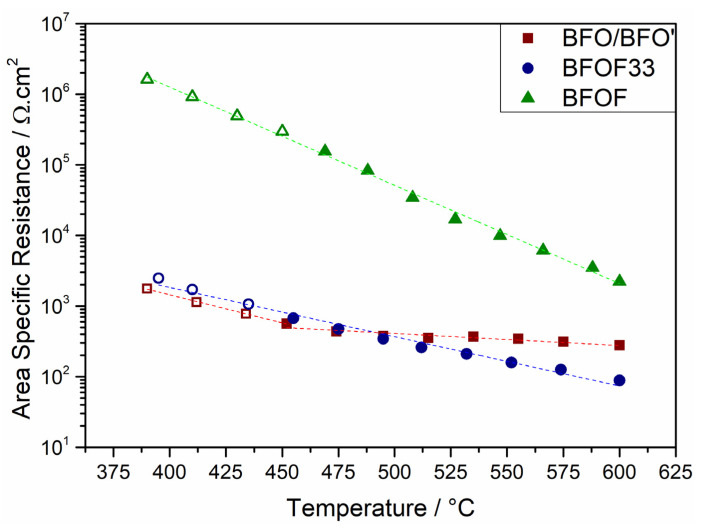
Logarithmic plot of the area specific resistance. Dashed fitting lines highlight the behavior in case of BFOF33 (●) and BFOF (▲). BFO/BFO’ (■) exhibits a bend trajectory. Hollow symbols represent the temperatures with open semicircles.

Figure 14 compares the area specific resistances of all three compounds. It is evident that BFO’ and BFOF33 show similar EIS responses, whereas BFOF exhibits much higher resistances by three orders of magnitude. The incorporation of oxygen at the electrode requires the presence of anion vacancies, which were below the detection limit by diffraction methods for cubic BaFeO_2_F [19]. This most likely also limits the generation of vacancies at the surface of the compound. Due to the nearly full occupation of the anion lattice and the pinning of the iron valency to a trivalent state, which limits electron transport, this process is strongly blocked. This well explains the difference of BFOF as compared to BFOF33 as well as BFO’.

**Figure 15:**
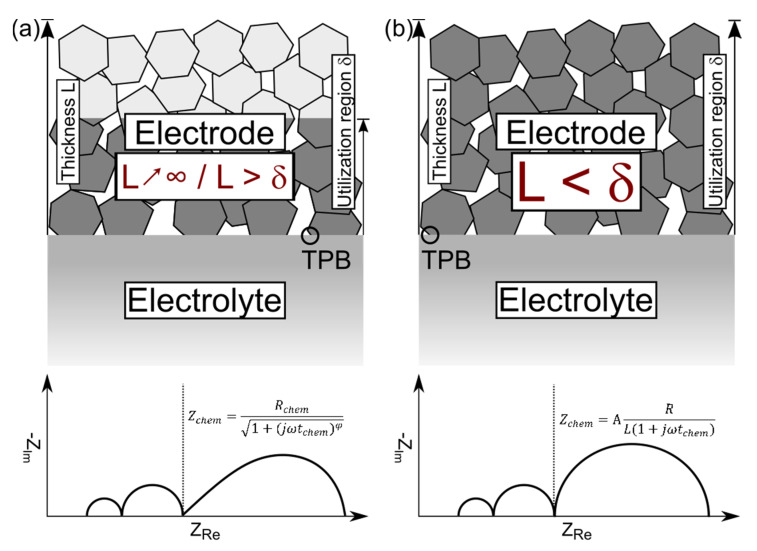
Schematic of the penetration of the utilization region δ into the electrode in dependency of its thickness L with the corresponding impedance models: (**a**) depicts the relation represented by Equation (2); (**b**) illustrates the case discussed for Equation (12).

The compounds BFO’ and BFOF33, which both possess a large amount of vacancies of c_V,oxygen_ ≈ 0.33 per Fe, exhibit only smaller conceptual differences regarding the electrochemical processes expressed by the differences found for the Bode plots. The Gerischer type model considers the oxygen uptake at the gas phase/electrode interface together with the bulk diffusion as the co-limiting factors in this process. Adler et al. [55] elaborated the case of a surface limitation due to an exceeding of the utilization zone throughout the whole electrode thickness. Figure 15 depicts the two special cases of the penetration of the utilization region. Further, it connects the mentioned limits with the mathematical description for Z_chem_ and representation inside a Nyquist plot. Adler et al. derived the solution for these boundary conditions in [55] for the equation:

(11) $$Z_{\mathrm{chem}}=\frac{R_{\mathrm{chem}}}{\sqrt{{1+j\omega t}_{\mathrm{chem}}}}\;|\mathrm{Tan}h\left(\frac{L}{\delta }\sqrt{{1+j\omega t}_{\mathrm{chem}}}\right)$$

with L as electrode thickness and δ the utilization region inside the electrode. For an infinite electrode thickness L, the term Tanh(L/δ) approaches unity, yielding Equation (2). As *δ* becomes larger than L, Equation (11) turns into a linear relation, which impedance is related to 1/L:

(12) $$Z_{\mathrm{chem}}=A\frac{R}{L\left(1+{j\omega t}_{\mathrm{chem}}\right)}$$

with A including thermodynamical and morphological parameters. In addition to the underlying theory of a utilization zone penetrating the electrode material, Figure 15 also takes into account the role of the triple phase boundary (TPB). The TPB was suggested to be the crucial region where the electrochemical process of oxygen uptake and dispersion happens. However, with the introduction of the Gerischer type impedance model, Adler et al. [52] extended the one dimensional TPB reaction into the electrode material in case of a porous mixed ionic-electronic conductor. Recent studies gave indication that the model of a spot-sized region is insufficient and does not capture the whole process [57]. The linear case results in a more semicircle like shape as depicted in Figure 15b. It may only occur if solid-state diffusion within the electrode is fast allowing for a utilization of the whole internal surface area for oxygen uptake limited by the film thickness L.

This behavior is in principle indicated for BFOF33 and might be interpreted as follows: The introduction of fluorine into the anion sub-lattice might provide a way to slightly enhance the oxygen ion mobility within the oxyfluoride lattice once a sufficient amount of vacancies is available. In a simple picture, F^−^ is a more mobile ion than O^2−^, and by this the fluoride ion might be able to allocate a vacancy to an oxide ion which is in the process of moving. Thus, the inner mobility of ions might be increased in BaFeO_2.33_F_0.33_▯_0.33_, and the electrode limiting process might be dominated by the fast ion diffusion and the utilization of the whole electrode surface according to the model elaborated by Adler et al. [55].

Evaluation of the area specific resistance in Figure 14 shows that the pure oxide BFO’ does not exhibit a linear Arrhenius type behavior, while the fluorinated films show Arrhenius type behavior, as indicated by the dashed lines. Within a VT-XRD study (*article in preparation*), it was found that BFO transforms to BFO’ under incorporation of O_2_ (hence oxidation) above 250 °C. The oxygen is then released above 500 °C and BFO’ is reversed to BFO. Hence, the kink and the unsteady trajectory at temperatures above 450 °C are a result of chemical processes related to the incorporation of oxygen and the resulting phase transformation. With this oxygen uptake, BFO’ becomes isotypic to BFOF33, and the linear behavior above 450 °C up to 590 °C agrees well with a fairly stable composition within this temperature range. The Gerischer related impedance response can in this case be interpreted as bulk diffusion limited, which is in agreement with the availability of high amounts of vacancies, the increase of amounts of Fe^4+^, which acts as holes for electron transport, and the fact that ionic re-arrangements are restricted to the less mobile O^2−^ ion only.

**Figure 16:**
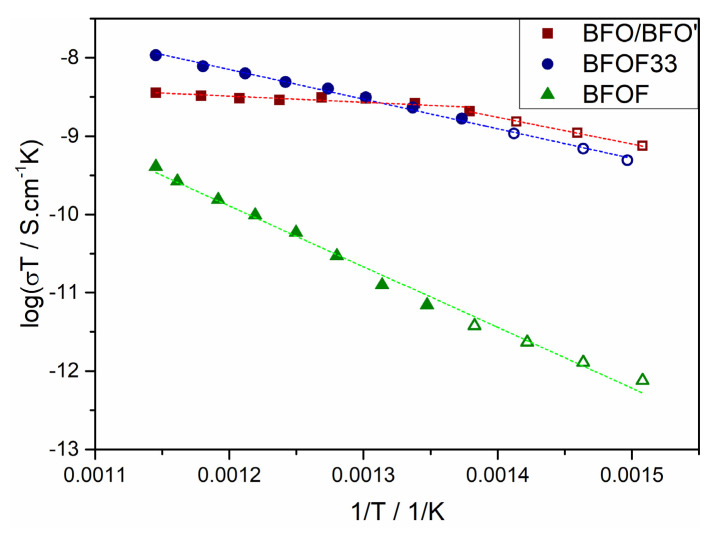
Arrhenius plot for the conductivity of BFO/BFO’ (■), BFOF33 (●) and BFOF (▲). Dashed lines represent the performed fit for the activation energy.

Table 2 lists the activation energies extracted from the Arrhenius plots shown in Figure 16 (note that two activation energies were extracted for BFO due to the apparent temperature dependent differences in composition). In the temperature range 390–470 °C the activation energies for BFO’ and BFOF33 appear to be similar (0.29 to 0.33 eV) and half of the value of BFOF (0.67 eV). Around 490 °C the conductivity of BFO levels and the activation energy drops down to 0.07 eV. Again, these differences in activation energies agree well with the conclusions described in the previous paragraph. Above 490 °C BFOF33 exhibits its highest conductivity reaching a value of 1∙10^−5^ S/cm at T = 575 °C, which is close to the decomposition temperature, while BFO and BFOF show values of 3∙10^−6^ S/cm and 2∙10^−7^ S/cm, respectively.

## 4. Conclusions

In this work, highly porous symmetrical films of BaFeO_2.67_, BaFeO_2.33_F_0.33_ and BaFeO_2_F were successfully synthesized on YSZ8 without the need of an intermediate layer. The porosity is achieved by spin coating nano-powder produced by the NSP process on YSZ substrates followed by a short sintering step. The as-prepared films feature a thickness of around 8 μm. The topochemical fluorination of the films is successfully performed by an interdiffusion and vapor approach for BaFeO_2.33_F_0.33_ and BaFeO_2_F, respectively. HTXRD revealed an upper temperature limit of 590 °C and 710 °C for BaFeO_2.33_F_0.33_ and BaFeO_2_F, respectively. The interdiffusion approach introduced in this work has proven to give an easy option to adjust a desired fluorine level in a thin film opening up topochemical fluorination for metastable oxyfluorides, which cannot be directly synthesized.

Recent studies have already revealed an enhancing effect of fluorine doping of oxide materials on the oxygen reduction and transportation [3,58,59]. A small amount of fluorine may lower the activation energy for the non-charge transfer processes, such as oxygen reduction or bulk transportation. In this study, we focused on the evaluation of the impedance data by applying a 3D model developed for porous MIEC cathodes. By this, it is indicated that the non-charge transfer process is limited by the oxygen uptake rather than by bulk diffusion as it is the case for the parent oxide. In addition, the fluorine can also help to stabilize the phase of the parent oxide, especially for Ba-rich compounds with large structural complexity. However, we have shown that it is of crucial importance to adjust the fluoride concentration to a compound specific content, as high fluorination degrees with high overall occupation of anion sites and absence of vacancies will have a detrimental effect on the overall cathode performance.

## Figures and Tables

**Table 1:** Lattice parameters and cell volume per formula unit Z of BaFeX_y_ (X = O, F) for the sintered and topochemically modified films.

**Films**							
**Compound**	**Composition**	**SG**	**a [Å]**	**b [Å]**	**c [Å]**	**β [°]**	**V_Z_ [Å^3^]**
BFO	BaFeO_2.5_	*P2_1_/c*	6.9751(5)	11.7237(9)	23.4553(15)	98.865(6)	67.682(8)
BFO’	BaFeO_2.67_	*P2_1_/m*	10.1185(8)	5.6431(5)	6.9425(6)	92.298(7)	66.016(9)
BFOF33	BaFeO_2.__33_F_0.33_	*P2_1_/m*	10.1450(8)	5.6877(5)	6.9792(6)	92.246(8)	67.067(9)
BFOF	BaFeO_2_F	*Pm3m*	4.0583(1)	-	-	-	66.839(7)
**Literature Data**							
**Reference**	**Composition**	**SG**	**a [Å]**	**b [Å]**	**c [Å]**	**β [°]**	**V_Z_ [Å^3^]**
[18]	BaFeO_2.5_	*P2_1_/c*	6.9753(1)	11.7281(2)	23.4507(4)	98.813(1)	67.692(2)
*article in prep.*	BaFeO_2.67_	*P2_1_/m*	10.1635(3)	5.6489(1)	6.9537(2)	92.064(2)	66.495(3)
[19]	BaFeO_2.33_F_0.33_	*P2_1_/m*	10.1059	5.7094	6.9770(1)	93.107(1)	66.995(2)
[22]	BaFeO_2_F	*Pm3>m*	4.05884(3)				66.866(1)

**Table 2:** Activation energies as calculated from the Arrhenius plots in Figure 14.

Activation Energy, E_a_	BFO/BFO’	BFOF33	BFOF	Ba_x_Sr_1−x_Co_0.8_Fe_0.2_O_3−d_ (0.3 ≤ x ≤ 0.7) [56]	SrCo_0.8_Fe_0.2_O_3−d_ [57]	La_2_NiO_4_ [52]
[eV]	0.07(1)/0.29(1) *	0.33(1)	0.67(2)	0.32–0.40	2.4–2.6	1.00

*: note that the extraction of an activation energy for BFO is difficult due to the structural instabilities (changes in oxygen content) beginning around 490 °C, thus a linear fit over the whole temperature range is not possible.
